# Association Between Neutrophil-to-Lymphocyte Ratio and Mortality Risk Among Patients With Hyperlipidemia Across Different Glycemic Status: A Longitudinal Cohort Study From NHANES 1999–2018

**DOI:** 10.31083/RCM46797

**Published:** 2026-03-09

**Authors:** Qingzhi Zhou, Yan Chen, Haonan Ju, Xin Zhao

**Affiliations:** ^1^Department of Cardiology, The Second Hospital of Dalian Medical University, 116023 Dalian, Liaoning, China

**Keywords:** NLR, hyperlipidemia, mortality, inflammation, NHANES

## Abstract

**Background:**

The neutrophil-to-lymphocyte ratio (NLR) has been linked as a marker of systemic inflammation to adverse outcomes in various metabolic diseases. However, the association of the NLR with mortality risk among patients with hyperlipidemia remains inconclusive. Thus, this research aimed to investigate whether the NLR is associated with mortality risk among individuals with hyperlipidemia and to examine how glycemic status influences this relationship.

**Methods:**

Weighted Cox regression, restricted cubic splines (RCS), and subgroup analyses were employed to evaluate the association between the NLR and mortality risk in patients with hyperlipidemia. Time-dependent receiver operating characteristic (ROC) analysis was conducted to assess the predictive accuracy for mortality risk.

**Results:**

In Model 3, individuals in the highest NLR quartile had a 40% higher risk of all-cause mortality (95% confidence interval (CI): 1.19–1.65; *p* for trend <0.001) and an 86% higher risk of cardiovascular mortality (95% CI: 1.28–2.68; *p* for trend <0.001) compared with those in the lowest quartile. Time-dependent ROC analysis confirmed the superior performance of the NLR in predicting cardiovascular mortality risk. A significant interaction between the NLR and diabetes mellitus (DM) was observed for both all-cause and cardiovascular mortality in the subgroup analyses. Given this finding, we further examined the association between the NLR and mortality, stratified by glycemic status. The results indicated that the association between the NLR and mortality was stronger among individuals with DM.

**Conclusions:**

An elevated NLR is closely associated with an increased risk of mortality among individuals with hyperlipidemia, and the presence of DM significantly strengthens this association.

## 1. Introduction

Hyperlipidemia is a highly prevalent metabolic disorder and an established contributor to atherosclerotic cardiovascular disease (ASCVD) [1, 2]. In the United States, approximately 38% of adults have at least borderline high total cholesterol (≥200 mg/dL), and 27.8% show increased levels of low-density lipoprotein cholesterol (LDL-C ≥130 mg/dL) [3]. Globally, elevated LDL-C was responsible for an estimated [4, 5] million deaths in 2020. This represents a 19% increase since 2010, thereby emphasizing the significant health burden of dyslipidaemia [3]. Notably, emerging evidence indicates that dyslipidemia is affecting younger populations with increasing frequency, potentially predisposing individuals to premature coronary events [2, 4, 5]. These trends highlight the need to improve risk stratification and preventive strategies in hyperlipidemic patients beyond traditional lipid measurements.

Mounting evidence implicates chronic low-grade inflammation in the pathogenesis of hyperlipidemia-related ASCVD [6, 7, 8]. It has been demonstrated that hyperlipidaemia can instigate an immune response that accelerates the formation of atherosclerotic plaque. For instance, neutrophils and other granulocytes have been observed to actively participate in the process of arterial inflammation and the progression of plaque [9, 10, 11]. Consistently, patients with cardiovascular disease often exhibit elevated inflammatory biomarkers such as high-sensitivity C-reactive protein (hs-CRP) and interleukin-6 [12], even when cholesterol levels are controlled. In this context, immune-cell indices have emerged as important markers of cardiovascular risk [13, 14, 15].

Systemic inflammatory indicators calculated based on a complete blood count (CBC), such as the neutrophil-to-lymphocyte ratio (NLR), platelet-to-lymphocyte ratio (PLR), and monocyte-to-lymphocyte ratio (MLR), can effectively reflect the association between these biomarkers and atherosclerotic lesions [16, 17]. NLR is characterized by its stability, easy accessibility, and low cost, and it can serve as an indicator reflecting the imbalance between innate and adaptive immunity [18]. Chen *et al*. [19] found that NLR levels above 3.42 were associated with 1.82-fold and 2.07-fold higher risks of all-cause and cardiovascular mortality, respectively, among individuals with pre-diabetes mellitus (DM) and DM. Furthermore, Hong *et al*. [20] also found a close association between an elevated NLR and adverse outcomes in patients with hypertension. However, limited evidence exists regarding the association between NLR and mortality risk in individuals with hyperlipidemia. Clarifying this relationship is essential for improving the clinical prognosis of these patients [21].

Notably, in metabolic conditions such as DM, interactions between inflammatory and insulin signaling pathways may jointly aggravate insulin resistance and endothelial dysfunction, thereby amplifying the contribution of inflammation to the progression of atherosclerosis and adverse cardiovascular outcomes [7, 22]. Given that patients with DM often have concurrent hyperlipidemia in primary care and clinical practice [23], particularly characterized by atherogenic dyslipidemia such as elevated triglyceride (TG) and small dense LDL particles that significantly raise cardiovascular risk [4, 23, 24], it is important to account for the potential influence of DM when evaluating the relationship between NLR and mortality risk in patients with hyperlipidemia.

In summary, this study, based on the National Health and Nutrition Examination Survey (NHANES) data, aims to evaluate the association of NLR with all-cause and cardiovascular mortality in patients with hyperlipidemia and to explore how this association varies across different glycemic status.

## 2. Materials and Methods

### 2.1 Study Population and Data Source

Data for this study were derived from the NHANES, a representative, stratified, multistage sampling program conducted in the United States [25]. Survival outcomes and death causes were identified using NHANES data linked with the National Death Index (NDI). Our study included data from NHANES surveys conducted between 1999 and 2018, initially comprising 101,316 participants. Individuals younger than 18 years (n = 42,112), those without survival status data (n = 140), without hyperlipidemia (n = 21,422), lacking inflammatory markers (n = 575), or missing covariate data (n = 23,997) were excluded. Consequently, 13,070 eligible participants were retained for analysis (**Supplementary Fig. 1**).

### 2.2 Definition of Hyperlipidemia

Hyperlipidemia was identified if participants met any of the following criteria: elevated LDL-C (≥130 mg/dL), total cholesterol (≥200 mg/dL), or triglycerides (≥150 mg/dL); reduced high-density lipoprotein cholesterol (HDL-C) (<40 mg/dL in men or <50 mg/dL in women); or reported use of lipid-lowering medications [26].

### 2.3 Study Variables

In this study, covariates were selected based on previous related studies and included demographic, socioeconomic, lifestyle, and biochemical factors [27, 28, 29]. The selected demographic and socioeconomic covariates comprised age, sex, ethnicity, education status, and socioeconomic status (assessed by poverty income ratio). Lifestyle-related factors consisted of smoking status and alcohol consumption categorized into heavy, moderate, mild, or never drinkers. Biochemical variables included fasting blood glucose (FBG), glycated hemoglobin (HbA1c), blood lipid profile (total cholesterol, triglycerides, LDL-C, and HDL-C), and hematological parameters such as white blood cell (WBC) count, neutrophil count, and lymphocyte count, as well as self-reported histories of cardiovascular disease, cancer, diabetes mellitus, and current usage of lipid-lowering medications. Detailed measurement protocols and further descriptions of these variables are available in the **Supplementary Table 1**.

### 2.4 Statistical Analysis

Categorical variables were summarized as frequencies with weighted percentages, and continuous variables were reported as medians with interquartile ranges (IQR). Kaplan-Meier curves and log-rank tests assessed differences in survival among NLR quartiles. To determine the association between NLR and mortality, weighted Cox proportional hazards regression models were used. The nonlinear association between NLR and mortality was evaluated using restricted cubic splines (RCS) with four knots. Time-dependent receiver operating characteristic (ROC) analyses assessed the predictive accuracy of NLR for mortality at 1, 3, 5, and 10 years by calculating areas under the curve (AUC). Subgroup analyses evaluated interactions by sex, age, ethnicity, alcohol use, smoking, cancer history, DM, and cardiovascular disease. R 4.2.2 (R Foundation for Statistical Computing, Vienna, Austria) was used for all analyses, and statistical significance was set at a *p*-value below 0.05.

## 3. Results

### 3.1 Participants Characteristics

A total of 13,070 adults with hyperlipidemia were analyzed, with a median age of 51 years and 51.17% being women (Table 1). Over a median follow-up of 10.7 years, 2265 deaths occurred, of which 617 were attributed to cardiovascular causes. Participants were divided into four groups according to baseline NLR levels. Compared with participants in the lowest NLR quartile, those with higher NLR levels were more likely to be older, male, non-Hispanic White, and current smokers, and to have higher levels of FBG, triglycerides, WBC, and neutrophils, as well as a greater prevalence of DM, cardiovascular disease (CVD), and cancer.

**Table 1.** **Baseline characteristics by NLR quartiles**.

Variables		Total (N = 13,070)	Q1 (0.009, 1.474)	Q2 (1.474, 1.944)	Q3 (1.944, 2.588)	Q4 (≥2.588)	*p*-value
Age (years)		51.00 (38.00, 63.00)	49.00 (35.00, 60.00)	50.00 (37.00, 61.00)	50.00 (38.00, 62.00)	55.00 (43.00, 68.00)	<0.001
Sex, n (%)							0.068
	Female	6594 (51.17)	1752 (53.77)	1661 (51.38)	1638 (51.16)	1543 (48.67)	
	Male	6476 (48.83)	1541 (46.23)	1553 (48.62)	1645 (48.84)	1737 (51.33)	
Race, n (%)							<0.001
	Non-Hispanic White	6382 (72.28)	1143 (60.62)	1498 (71.34)	1714 (75.28)	2027 (80.53)	
	Non-Hispanic Black	2285 (8.88)	978 (16.49)	519 (8.13)	443 (6.71)	345 (5.02)	
	Mexican American	2325 (7.76)	588 (9.32)	628 (8.48)	618 (7.46)	491 (5.98)	
	Other Race	2078 (11.08)	584 (13.58)	569 (12.05)	508 (10.55)	417 (8.47)	
Education levels, n (%)							0.005
	<high school	3517 (16.69)	875 (16.60)	852 (16.13)	905 (16.63)	885 (17.35)	
	=high school	3097 (24.52)	744 (22.36)	789 (24.84)	732 (23.09)	832 (27.59)	
	>high school	6456 (58.79)	1674 (61.04)	1573 (59.03)	1646 (60.28)	1563 (55.06)	
PIR, n (%)							0.003
	<1	2485 (12.57)	660 (13.35)	580 (11.56)	623 (12.63)	622 (12.82)	
	1–3	5519 (35.91)	1372 (37.16)	1298 (32.87)	1409 (36.74)	1440 (36.94)	
	>3	5066 (51.51)	1261 (49.49)	1336 (55.57)	1251 (50.63)	1218 (50.24)	
Smoking status, n (%)							<0.001
	Never	6732 (51.90)	1861 (56.52)	1744 (53.91)	1692 (53.19)	1435 (44.53)	
	Former	3628 (27.39)	820 (25.86)	844 (26.63)	901 (26.05)	1063 (30.86)	
	Current	2710 (20.71)	612 (17.62)	626 (19.46)	690 (20.76)	782 (24.61)	
Alcohol consumption, n (%)							0.003
	Never	1789 (10.56)	499 (11.75)	432 (10.57)	449 (9.99)	409 (10.08)	
	Former	2516 (16.11)	556 (14.13)	591 (14.48)	650 (16.51)	719 (19.05)	
	Mild	4552 (38.11)	1163 (38.25)	1077 (37.78)	1123 (37.65)	1189 (38.78)	
	Moderate	1808 (16.28)	466 (16.94)	493 (17.80)	450 (16.68)	399 (13.81)	
	Heavy	2405 (18.94)	609 (18.93)	621 (19.37)	611 (19.16)	564 (18.28)	
BMI (kg/m^2^)		28.60 (25.11, 33.14)	27.90 (24.80, 32.20)	28.53 (25.20, 32.79)	29.03 (25.40, 33.80)	28.73 (25.16, 33.70)	<0.001
DM, n (%)							<0.001
	No	10,201 (81.56)	2669 (85.76)	2578 (84.68)	2542 (80.64)	2412 (75.70)	
	Yes	2869 (18.44)	624 (14.24)	636 (15.32)	741 (19.36)	868 (24.30)	
CVD, n (%)							<0.001
	No	11,333 (88.65)	2978 (91.80)	2875 (90.95)	2852 (89.22)	2628 (83.01)	
	Yes	1737 (11.35)	315 (8.20)	339 (9.05)	431 (10.78)	652 (16.99)	
Cancer, n (%)							<0.001
	No	11,719 (89.07)	3032 (90.80)	2915 (90.20)	2966 (89.34)	2806 (86.14)	
	Yes	1351 (10.93)	261 (9.20)	299 (9.80)	317 (10.66)	474 (13.86)	
Lipid-lowering drugs, n (%)							<0.001
	No	9763 (74.31)	2618 (80.46)	2466 (75.95)	2474 (76.00)	2205 (65.52)	
	Yes	3307 (25.69)	675 (19.54)	748 (24.05)	809 (24.00)	1075 (34.48)	
Laboratory data							
	HbA1c (%)	5.50 (5.20, 5.80)	5.50 (5.20, 5.80)	5.50 (5.20, 5.80)	5.50 (5.20, 5.80)	5.50 (5.20, 5.90)	0.030
	FBG (mmol/L)	5.61 (5.22, 6.11)	5.55 (5.16, 6.00)	5.55 (5.18, 6.05)	5.61 (5.22, 6.16)	5.72 (5.27, 6.36)	<0.001
	TC (mmol/L)	5.30 (4.50, 5.92)	5.40 (4.65, 6.00)	5.35 (4.63, 5.95)	5.33 (4.55, 5.97)	5.09 (4.22, 5.79)	<0.001
	HDL-C (mmol/L)	1.27 (1.06, 1.58)	1.29 (1.06, 1.63)	1.29 (1.06, 1.58)	1.24 (1.03, 1.55)	1.24 (1.03, 1.53)	<0.001
	TG (mmol/L)	1.38 (0.97, 1.95)	1.33 (0.93, 1.93)	1.37 (0.96, 1.95)	1.41 (1.02, 2.00)	1.38 (0.97, 1.91)	0.011
	LDL-C (mmol/L)	3.21 (2.53, 3.78)	3.31 (2.64, 3.83)	3.26 (2.64, 3.80)	3.26 (2.59, 3.80)	3.00 (2.28, 3.65)	<0.001
	WBC (10^9^/L)	6.60 (5.50, 8.00)	5.80 (4.90, 7.00)	6.20 (5.30, 7.40)	6.70 (5.80, 8.00)	7.50 (6.30, 9.10)	<0.001
	Neutrophils (10^9^/L)	3.80 (3.00, 4.80)	2.70 (2.20, 3.40)	3.40 (2.90, 4.10)	4.10 (3.50, 4.90)	5.20 (4.20, 6.30)	<0.001
	Lymphocytes (10^9^/L)	1.90 (1.60, 2.40)	2.30 (2.00, 2.90)	2.00 (1.70, 2.40)	1.90 (1.60, 2.20)	1.50 (1.30, 1.90)	<0.001

Data are presented as median (IQR) or n (%). Abbreviations: NLR, neutrophil-lymphocyte ratio; PIR, poverty income ratio; BMI, body mass index; DM, diabetes mellitus; CVD, cardiovascular disease; HbA1c, glycated hemoglobin; FBG, fasting blood glucose; TC, total cholesterol; HDL-C, high-density lipoprotein cholesterol; TG, triglyceride; LDL-C, low-density lipoprotein cholesterol; WBC, white blood cell.

### 3.2 Kaplan-Meier Survival Analysis 

Kaplan-Meier survival curves were constructed to assess differences in survival among NLR quartiles (**Supplementary Fig. 2**). Notable differences in all-cause and cardiovascular mortality were observed among NLR quartiles. Survival probability decreased progressively with increasing NLR quartiles.

### 3.3 Associations Between NLR and Mortality Risk

**Fig. 1.**
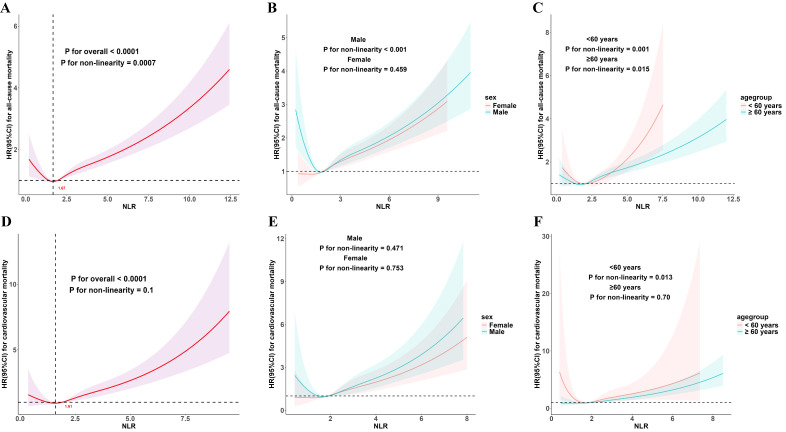
**Dose–response association of NLR with mortality in hyperlipidemic patients**. Dose-response relationships of NLR with all-cause mortality in the overall population (A), sex subgroups (B), and age subgroups (C); and with cardiovascular mortality in the overall population (D), sex subgroups (E), and age subgroups (F). Red numbers indicate NLR threshold points. Solid lines denote HR estimates, with shaded areas representing 95% CIs. Adjustments were consistent with those applied in Model 3.

Weighted Cox regression analysis showed that in Model 3, higher NLR was significantly associated with increased risks of all-cause (hazard ratio (HR) 1.40, 95% confidence interval (CI) 1.19–1.65) and cardiovascular mortality (HR 1.86, 95% CI 1.28–2.68) (Table 2). In addition, after log-transforming NLR, its associations with mortality were similar to those observed in the primary analysis (**Supplementary Table 2**). RCS analysis showed a ‘J’-shaped relationship between NLR and mortality among individuals with hyperlipidemia. Specifically, risk elevations became evident when NLR surpassed threshold points (all-cause mortality threshold at NLR = 1.67; cardiovascular mortality threshold at NLR = 1.61). Additionally, similar dose-response patterns were observed in subgroups stratified by sex and age (Fig. 1).

**Table 2.** **Association between NLR and mortality among patients with hyperlipidemia**.

Variable		Events	Model 1		Model 2		Model 3	
			HR (95% CI)	*p*-value	HR (95% CI)	*p*-value	HR (95% CI)	*p*-value
All-cause mortality								
	NLR Q1	385	REF		REF		REF	
	NLR Q2	424	1.04 (0.84–1.29)	0.705	1.02 (0.83–1.25)	0.837	0.99 (0.81–1.22)	0.955
	NLR Q3	567	1.23 (1.02–1.48)	0.033	1.01 (0.85–1.20)	0.892	0.93 (0.78–1.10)	0.375
	NLR Q4	889	2.45 (2.10–2.86)	<0.001	1.54 (1.31–1.80)	<0.001	1.40 (1.19–1.65)	<0.001
	*p* for trend		<0.001		<0.001		<0.001	
Cardiovascular mortality								
	NLR Q1	89	REF		REF		REF	
	NLR Q2	105	1.00 (0.62–1.61)	0.995	1.03 (0.66–1.60)	0.909	0.98 (0.64–1.51)	0.933
	NLR Q3	157	1.57 (1.01–2.42)	0.044	1.32 (0.89–1.97)	0.168	1.19 (0.80–1.79)	0.395
	NLR Q4	266	3.47 (2.42–4.97)	<0.001	2.07 (1.47–2.90)	<0.001	1.86 (1.28–2.68)	0.001
	*p* for trend		<0.001		<0.001		<0.001	

Note: Model 1: unadjusted; Model 2: adjusted for age and sex; Model 3: further adjusted for BMI, education, race, PIR, smoking, alcohol use, CVD, cancer, DM, and lipid-lowering medication use. Abbreviations: HR, hazard ratio; CI, confidence interval; REF, reference.

### 3.4 Time-Dependent ROC Curves of the NLR for Predicting All-Cause and Cardiovascular Mortality

**Fig. 2.**
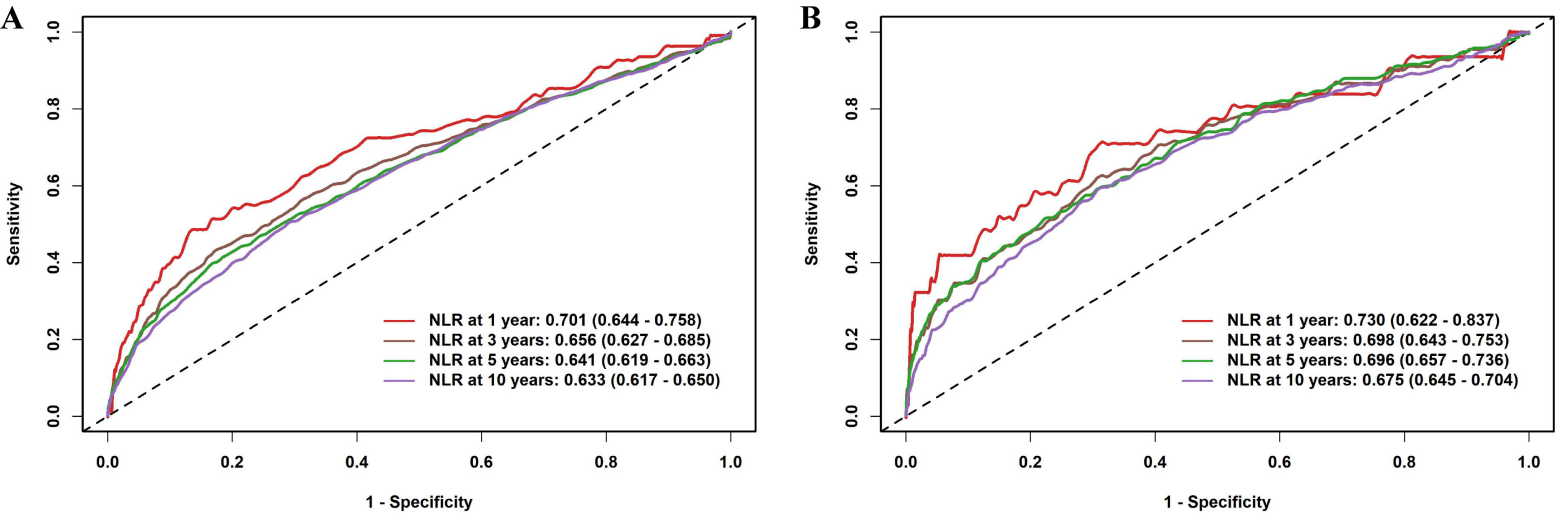
**Time-dependent ROC curves of the NLR for predicting mortality outcomes**. (A) All-cause mortality; (B) Cardiovascular mortality. Abbreviations: ROC, receiver operating characteristic.

Time-dependent ROC analyses were performed to determine the prognostic capability of NLR at different time intervals (Fig. 2). The time-dependent ROC analysis showed that the AUCs for NLR in predicting cardiovascular mortality were consistently higher than those for all-cause mortality across 1-, 3-, 5-, and 10-year follow-ups. **Supplementary Fig. 3** shows the trends over time in the AUC of NLR for predicting mortality risk.

### 3.5 Association Between NLR and Mortality Among Patients With Hyperlipidemia According to Glycemic Status

**Fig. 3.**
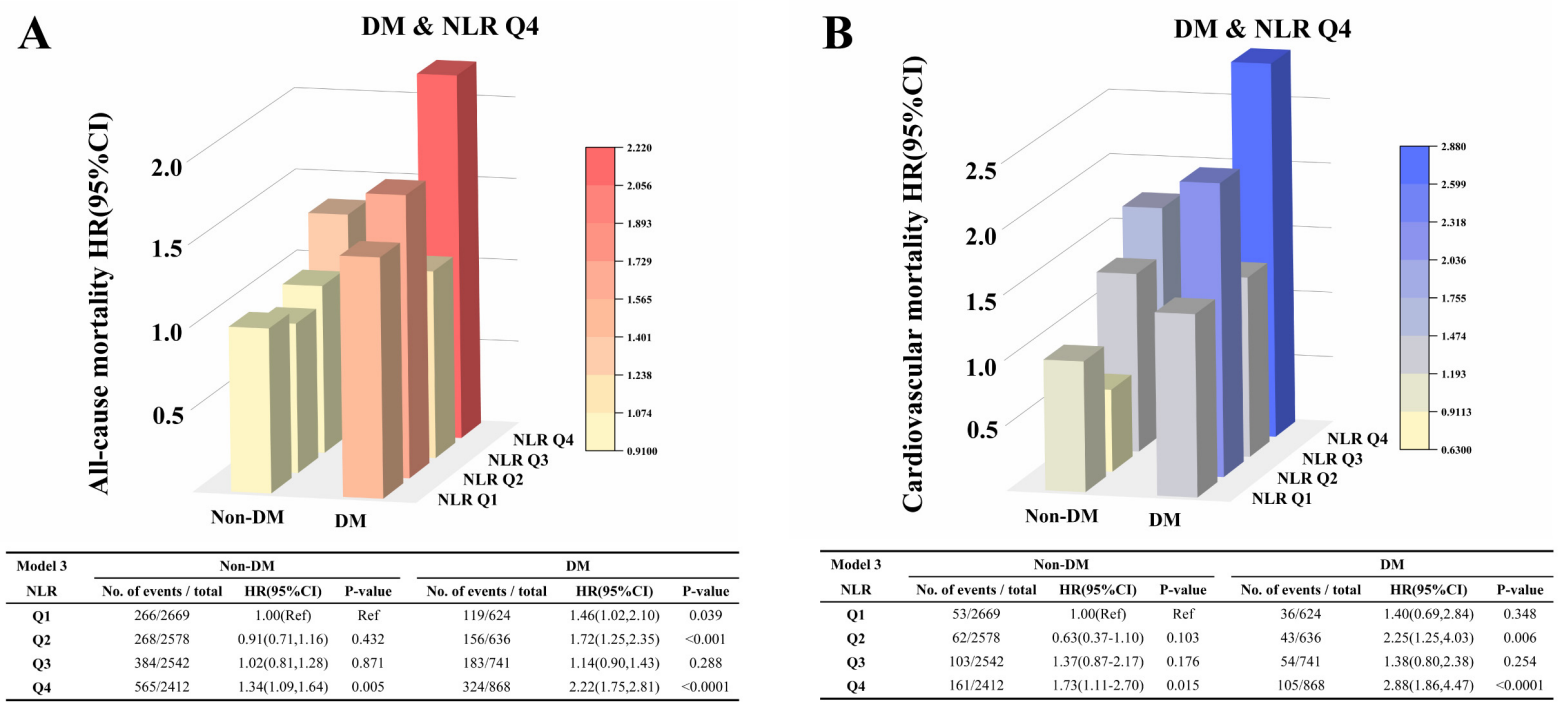
**Association between NLR and mortality risk among patients with hyperlipidemia according to glycemic status**. (A) All-cause mortality; (B) Cardiovascular mortality.

Subgroup analyses revealed significant interactions between DM and NLR quartiles for both all-cause (*p* for interaction = 0.04) and cardiovascular mortality (*p* for interaction = 0.01) (**Supplementary Figs. 4,5**). Stratified analyses by glycemic status showed that participants with DM and NLR in the highest quartile (Q4) had the greatest mortality risk, with a 2.22-fold higher risk of all-cause mortality (HR = 2.22; 95% CI: 1.75–2.81; *p* < 0.0001) and a 2.88-fold higher risk of cardiovascular mortality (HR = 2.88; 95% CI: 1.86–4.47; *p* < 0.0001) compared with those without DM in the lowest NLR quartile (Q1) (Fig. 3). These findings highlight that the association between elevated NLR and mortality risk is stronger in individuals with DM.

## 4. Discussion

In this longitudinal cohort study, we found that NLR was positively associated with both all-cause and cardiovascular mortality among patients with hyperlipidemia, and this association was stronger under DM status. Additionally, the time-dependent ROC analysis showed that NLR had a greater advantage in predicting cardiovascular mortality than in predicting all-cause mortality.

A previous study has shown that among individuals with metabolic syndrome, higher NLR is associated with a significantly increased mortality risk, with each unit increase linked to a 14% higher all-cause mortality and the highest quartile demonstrating approximately 1.5-fold greater risk compared to the lowest [30]. Similarly, in patients with type 2 diabetes, elevated NLR independently predicted approximately two-fold higher all-cause mortality and nearly three-fold higher cardiovascular mortality when comparing the highest with the lowest NLR quartiles [31]. Even in the general population, extreme NLR elevations (≥6) have been associated with approximately two-fold increased long-term mortality compared to normal NLR levels [32]. In this study, elevated NLR was positively associated with all-cause and cardiovascular mortality among patients with hyperlipidemia, consistent with evidence supporting NLR as a robust predictor of adverse metabolic diseases. Drechsler’s research suggests hyperlipidemia disrupts neutrophil homeostasis by altering their production, clearance, and mobilization, leading to elevated neutrophil counts in circulation and peripheral blood [21]. Within the NLR metric, neutrophils exert prominent pro-atherogenic effects, whereas lymphocytes have protective anti-atherogenic roles [33]. Neutrophils drive inflammation by initiating apoptosis and producing apoptotic debris, promoting lipid pool formation and thin-cap fibroatheroma plaque development, thus elevating cardiovascular risk [34]. Elevated lymphocyte counts reflect a balanced immune response and suppression of inflammatory pathways, thus protecting arterial vessels from the onset and progression of atherosclerosis [35]. The NLR partially represents the balance between pro- and anti-atherogenic processes, potentially explaining the heightened risk of cardiovascular events and mortality in hyperlipidemic patients. The strong association between high NLR and mortality is biologically plausible given that NLR integrates two critical dimensions of the immune response. Specifically, neutrophils are key effectors of innate inflammation, while lymphocytes (especially T-cells) orchestrate adaptive immunity; thus, an elevated NLR signifies excessive neutrophil-driven inflammation coupled with relative lymphopenia [36, 37]. This imbalance can lead to a state of chronic, low-grade inflammation alongside impaired immune regulation.

Interestingly, the results of subgroup analysis and interaction testing indicated an interaction between NLR and DM, which subsequently influenced the strength of the association between NLR and outcome events. Further analysis suggested that the joint effect of high NLR (Q4) and DM can synergistically increase the risk of all-cause and cardiovascular mortality among patients with hyperlipidemia. Both high lipid levels and hyperglycemia are known to trigger the release of inflammatory cytokines like interleukin-6 and tumor necrosis factor-α [38]. These cytokines not only induce oxidative stress and endothelial dysfunction (thereby accelerating atherosclerosis) [39, 40], but also interfere with insulin signaling, promoting insulin resistance. Therefore, when hyperlipidemia and DM coexist, the pro-atherogenic effects of inflammation and the degree of insulin resistance become further aggravated. Indeed, a clinical study has noted that NLR correlates positively with insulin resistance in diabetics [41], supporting a vicious cycle whereby inflammation and impaired metabolism fuel one another. Additionally, the specific interaction between inflammatory signaling pathways and insulin signaling pathways leads to enhanced metabolic insulin resistance and endothelial dysfunction [7, 22], exacerbating endothelial damage and consequently increasing the risk of target organ damage and mortality [42]. This may partially explain why higher NLR patients with DM exhibited a significantly increased risk of death.

## 5. Limitations

This study has several limitations. First, due to the observational nature of the NHANES dataset, a causal relationship between elevated NLR levels and mortality outcomes cannot be definitively established. Second, although extensive covariate adjustments were performed, the possibility of residual confounding from unmeasured or unknown factors, such as dietary patterns, physical activity, and additional inflammatory biomarkers, cannot be completely ruled out. Third, because NLR was measured only once at baseline, we were unable to assess the potential impact of longitudinal changes in NLR on mortality risk. Fourth, the lack of continuous hs-CRP measurements in the NHANES database precluded a direct comparison between NLR and hs-CRP in evaluating mortality risk among patients with hyperlipidemia. Finally, this study primarily focused on the association between NLR and mortality rather than its clinical application in risk prediction; therefore, further studies are warranted to evaluate the clinical utility of NLR in clinical settings.

## 6. Conclusions

In summary, our study found that elevated NLR was positively associated with both all-cause and cardiovascular mortality among patients with hyperlipidemia. Importantly, the observed interaction between NLR and DM highlights a clinically significant subgroup at particularly elevated risk, suggesting that dysregulated glucose metabolism may amplify the adverse effects of chronic inflammation. These findings provide compelling evidence to consider NLR as a practical and effective clinical biomarker, underscoring the potential utility of integrated therapeutic approaches targeting inflammation and glucose metabolism to mitigate mortality risk in individuals with hyperlipidemia.

## Availability of Data and Materials

Data for this study are freely available on the NHANES website at https://www.cdc.gov/nchs/nhanes/.

## Supplementary Material

Supplementary material associated with this article can be found, in the online version, at https://doi.org/10.31083/RCM46797.
