# Supplementary material for: Association Between Neutrophil-to-Lymphocyte Ratio and Mortality Risk Among Patients With Hyperlipidemia Across Different Glycemic Status: A Longitudinal Cohort Study From NHANES 1999–2018
Source: Rev Cardiovasc Med. 2026 Mar 9;27(3):46797. doi: 10.31083/RCM46797 (PMC13036540; doi:10.31083/RCM46797)
Supplement: Supplementary file 1 [file 2153-8174-27-3-46797-s1.zip › Supplementary Material.pdf]

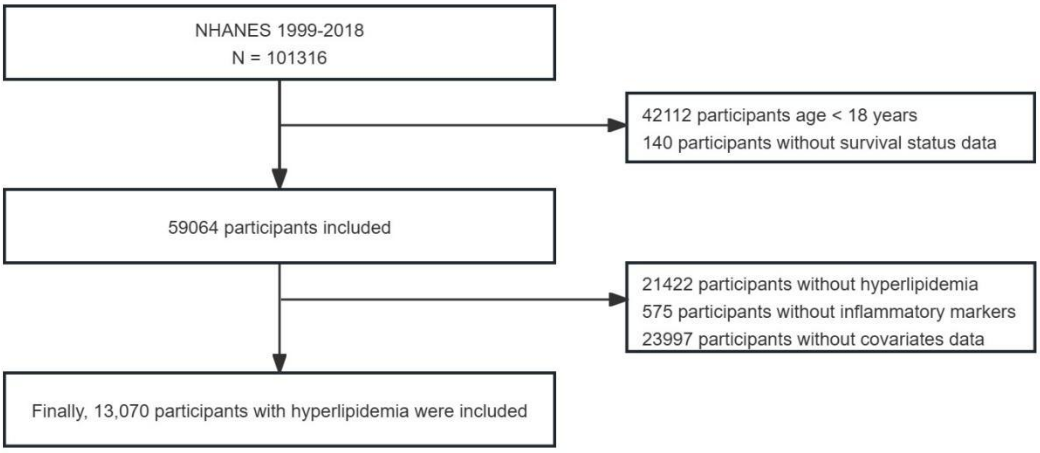

Supplementary Fig. 1 Flowchart for screening participants with hyperlipidemia from NHANES 1999-2018

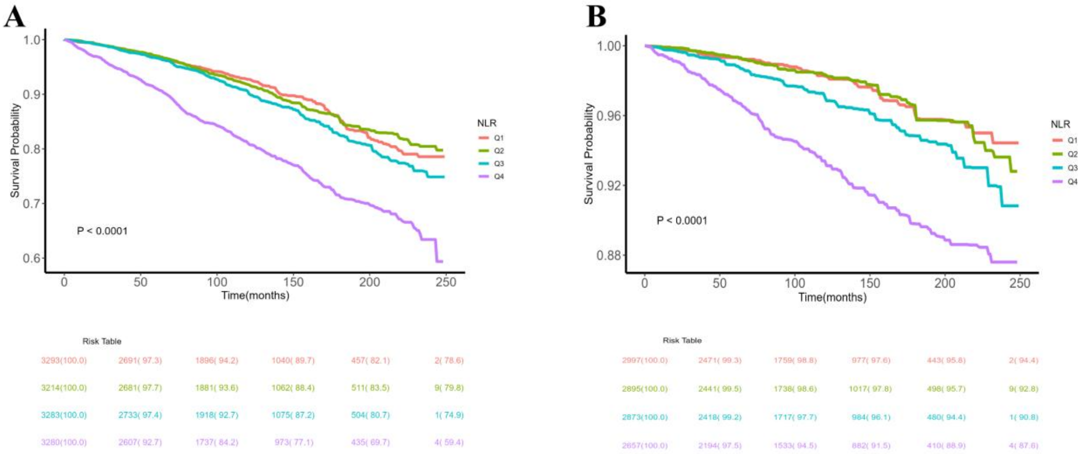

Supplementary Fig. 2 Kaplan-Meier survival curves and the number (percentage) of hyperlipidemic patients at risk across NLR quartiles A all-cause mortality; **B** cardiovascular mortality

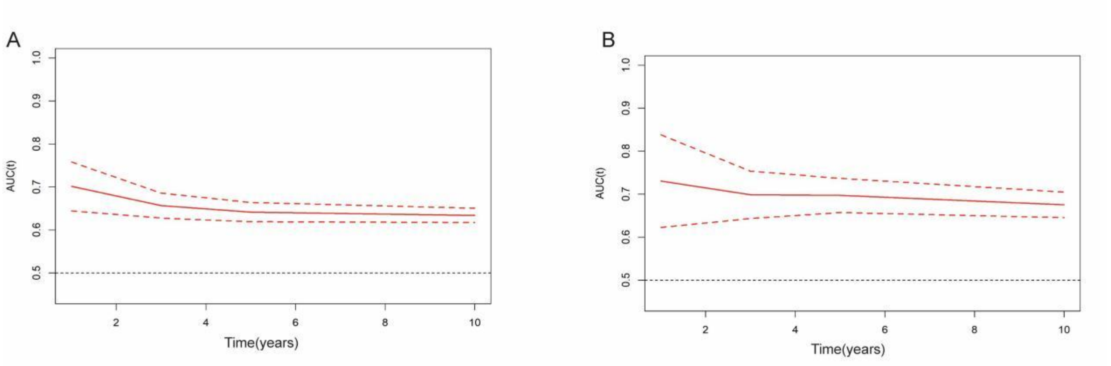

Supplementary Fig. 3 Time-dependent AUC values of the NLR for predicting all-cause mortality (A) and cardiovascular mortality (B)

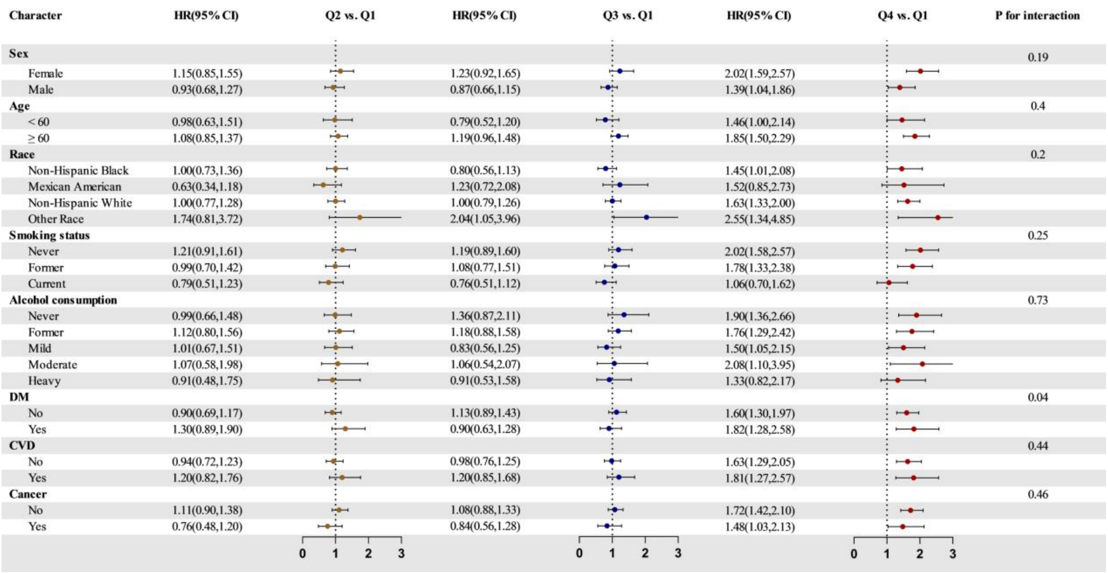

Supplementary Fig. 4 Subgroup analysis of the association between NLR and all-cause mortality

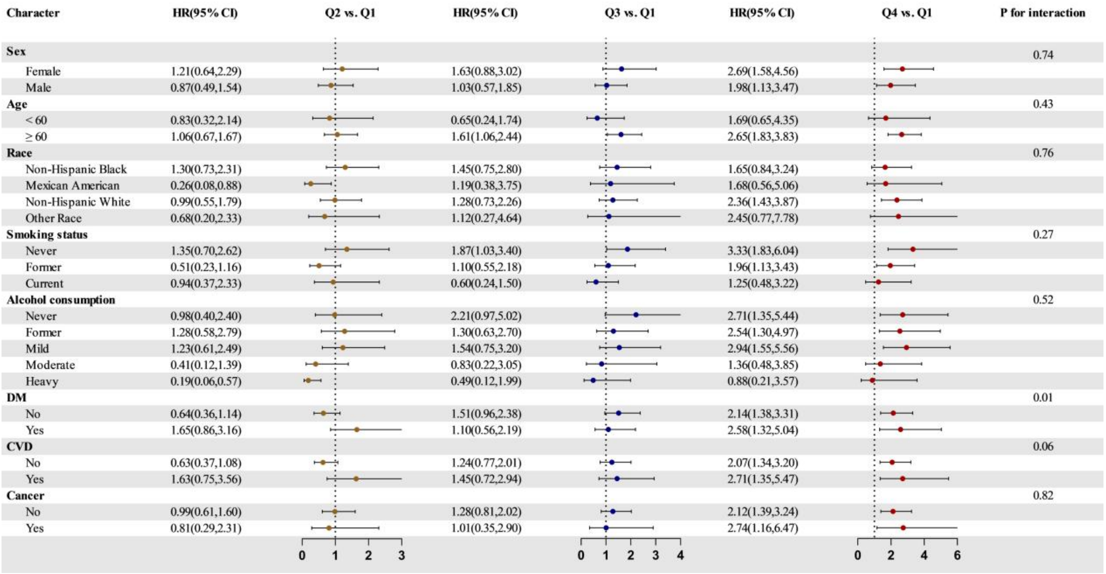

Supplementary Fig. 5 Subgroup analysis of the association between NLR and cardiovascular mortality

Supplementary Table 1 Covariates definitions

Variable	Definition	Reference
**Smoking status**	The classification of smoking status is as follows: never smokers (individuals who have smoked less than 100 cigarettes in their lifetime), former smokers (individuals who have smoked in the past but have quit smoking now), and current smokers individuals who have smoked at least 100 cigarettes in their lifetime and are still smoking now)	PMID: 29537517
**Alcohol consumption**	Heavy drinking is defined as consuming ≥ 3 drinks per day for women, ≥ 4 drinks per day for men, or engaging in binge drinking on ≥ 5 days per month; moderate drinking is characterized by consuming two drinks per day for females, three drinks per day for males, or binge drinking on ≥ 2 days per month; mild drinking is designated for those who do not meet the criteria for heavy or moderate drinking, while never drinking refers to individuals who have consumed < 12 drinks in their lifetime	PMID: 34558851
**Diabetes mellitus**	Diabetes mellitus was defined as having a self-reported physician diagnosis, fasting plasma glucose levels ≥ 7.0 mmol/L, glycosylated hemoglobin (HbA1c) levels ≥ 6.5%, random blood glucose levels ≥ 11.1 mmol/L, 2-hour plasma glucose levels ≥ 11.1 mmol/L during a 75-gram oral glucose tolerance test (OGTT), and/or current use of antidiabetic medications.	PMID: 33168652
**Cardiovascular Disease**	The history of CVD was based on self reported physician diagnoses obtained during individual interviews using a standardized medical conditions questionnaire. Participants were asked: “Has a doctor or other health professional ever told you that you have congestive heart failure, coronary heart disease, angina, myocardial infarction, or stroke?” Participants who answered “yes” to any of these conditions were classified as having CVD.	PMID: 37328831
**Cancer**	The history of cancer was determined based on the results of a questionnaire survey	PMID: 39011047

Supplementary Table 2 Association between lnNLR and mortality among patients with hyperlipidemia

Variable	Events	Model 1		Model 2		Model 3	
		HR(95%CI)	P-value	HR(95%CI)	P-value	HR(95%CI)	P-value
**All-cause mortality**							
lnNLR Q1	385	REF		REF		REF	
lnNLR Q2	428	1.04(0.83-1.28)	0.75	1.01(0.83-1.24)	0.90	0.99(0.80-1.21)	0.90
lnNLR Q3	569	1.25(1.04-1.50)	0.02	1.03(0.86-1.22)	0.77	0.94(0.79-1.11)	0.46
lnNLR Q4	883	2.44(2.10-2.85)	<0.0001	1.53(1.31-1.80)	<0.0001	1.40(1.18-1.64)	<0.0001
P for trend		<0.0001		<0.0001		<0.0001	
**Cardiovascular mortality**							
lnNLR Q1	89	REF		REF		REF	
lnNLR Q2	106	0.98(0.61-1.58)	0.95	1.01(0.65-1.57)	0.98	0.96(0.63-1.48)	0.87
lnNLR Q3	156	1.58(1.02-2.45)	0.04	1.34(0.90-1.98)	0.15	1.20(0.80-1.80)	0.37
lnNLR Q4	266	3.48(2.43-5.00)	<0.0001	2.08(1.48-2.92)	<0.0001	1.87(1.29-2.70)	<0.001
P for trend		<0.0001		<0.0001		<0.001	

Note: Model 1: unadjusted; Model 2: adjusted for age and sex; Model 3: further adjusted for BMI, education, race, PIR, smoking, alcohol use, CVD, cancer, DM, and lipid-lowering medication use.

Abbreviations: HR, hazard ratio**;** CI, confidence interval; REF, reference; NLR, neutrophil-lymphocyte ratio; BMI, body mass index; PIR, poverty income ratio; CVD, cardiovascular disease; DM, diabetes mellitus.
